# Healthcare Workers Bioresource: Study outline and baseline characteristics of a prospective healthcare worker cohort to study immune protection and pathogenesis in COVID-19

**DOI:** 10.12688/wellcomeopenres.16051.2

**Published:** 2020-10-12

**Authors:** João B Augusto, Katia Menacho, Mervyn Andiapen, Ruth Bowles, Maudrian Burton, Sophie Welch, Anish N Bhuva, Andreas Seraphim, Corinna Pade, George Joy, Melanie Jensen, Rhodri H Davies, Gabriella Captur, Marianna Fontana, Hugh Montgomery, Ben O’Brien, Aroon D Hingorani, Teresa Cutino-Moguel, Áine McKnight, Hakam Abbass, Mashael Alfarih, Zoe Alldis, Georgina L Baca, Alex Boulter, Olivia V Bracken, Natalie Bullock, Nicola Champion, Carmen Chan, Xose Couto-Parada, Keenan Dieobi-Anene, Karen Feehan, Gemma Figtree, Melanie C Figtree, Malcolm Finlay, Nasim Forooghi, Joseph M Gibbons, Peter Griffiths, Matt Hamblin, Lee Howes, Ivie Itua, Meleri Jones, Victor Jardim, Vikas Kapil, Wing-Yiu Jason Lee, Vineela Mandadapu, Celina Mfuko, Oliver Mitchelmore, Susana Palma, Kush Patel, Steffen E Petersen, Brian Piniera, Rosalind Raine, Alicja Rapala, Amy Richards, Genine Sambile, Jorge Couto de Sousa, Michelle Sugimoto, George D Thornton, Jessica Artico, Dan Zahedi, Ruth Parker, Mathew Robathan, Lauren M Hickling, Ntobeko Ntusi, Amanda Semper, Tim Brooks, Jessica Jones, Art Tucker, Jessry Veerapen, Mohit Vijayakumar, Theresa Wodehouse, Lucinda Wynne, Thomas A Treibel, Mahdad Noursadeghi, Charlotte Manisty, James C Moon

**Affiliations:** 1 Institute of Cardiovascular Science, University College London, London, UK; 2 Barts Heart Centre, St Bartholomew's Hospital, Barts Health NHS Trust, London, UK, London, UK; 3 Centre for Cardiovascular Medicine and Devices, William Harvey Research Institute, Queen Mary University of London, London, UK; 4 William Harvey Research Institute, Queen Mary University of London, London, UK; 5 NIHR Cardiovascular Biomedical Research Unit, St Bartholomew's Hospital, Barts Health NHS Trust, London, UK; 6 The Blizard Institute, Queen Mary University of London School of Medicine and Dentistry, London, UK; 7 Royal Free London NHS Foundation Trust, London, UK; 8 MRC Unit for Lifelong Health and Ageing, University College London, London, UK; 9 Centre for Human Health and Performance, University College London, London, UK; 10 Department of Virology, Barts Health NHS Trust, London, UK; 11 Division of Medicine, University College London, London, UK; 12 Royal North Shore Hospital; The University of Sydney, Sydney, Australia; 13 School of Health Sciences, University of Southampton & NIHR Applied Research Collaboration (ARC), Wessex, UK; 14 Wolfson Institute of Preventative Medicine, Centre for Cancer Prevention, Queen Mary University of London, London, UK; 15 Department of Applied Health Research, University College London, London, UK; 16 School of Clinical Medicine, University of Cambridge, Cambridge, UK; 17 East London NHS Foundation Trust Unit for Social and Community Psychiatry, Newham Centre for Mental Health, London, UK; 18 Department of Medicine, University of Cape Town and Groote Schuur Hospital, Cape Town, South Africa; 19 Public Health England, Porton Down, UK; 20 Division of Infection and Immunity, University College London, London, UK

**Keywords:** COVID-19, healthcare workers, pandemic

## Abstract

**Background**: Most biomedical research has focused on sampling COVID-19 patients presenting to hospital with advanced disease, with less focus on the asymptomatic or paucisymptomatic. We established a bioresource with serial sampling of health care workers (HCWs) designed to obtain samples before and during mainly mild disease, with follow-up sampling to evaluate the quality and duration of immune memory.

**Methods**: We conducted a prospective study on HCWs from three hospital sites in London, initially at a single centre (recruited just prior to first peak community transmission in London), but then extended to multiple sites 3 weeks later (recruitment still ongoing, target n=1,000). Asymptomatic participants attending work complete a health questionnaire, and provide a nasal swab (for SARS-CoV-2 RNA by RT-PCR tests) and blood samples (mononuclear cells, serum, plasma, RNA and DNA are biobanked) at 16 weekly study visits, and at 6 and 12 months.

**Results**: Preliminary baseline results for the first 731 HCWs (400 single-centre, 331 multicentre extension) are presented. Mean age was 38±11 years; 67% are female, 31% nurses, 20% doctors, and 19% work in intensive care units. COVID-19-associated risk factors were: 37% black, Asian or minority ethnicities; 18% smokers; 13% obesity; 11% asthma; 7% hypertension and 2% diabetes mellitus. At baseline, 41% reported symptoms in the preceding 2 weeks. Preliminary test results from the initial cohort (n=400) are available: PCR at baseline for SARS-CoV-2 was positive in 28 of 396 (7.1%, 95% CI 4.9-10.0%) and 15 of 385 (3.9%, 2.4-6.3%) had circulating IgG antibodies.

**Conclusions**: This COVID-19 bioresource established just before the peak of infections in the UK will provide longitudinal assessments of incident infection and immune responses in HCWs through the natural time course of disease and convalescence. The samples and data from this bioresource are available to academic collaborators by application https://covid-consortium.com/application-for-samples/.

## Introduction

The global pandemic of severe acute respiratory syndrome coronavirus 2 (SARS-CoV-2) has led to more than 6 million infections and 300,000 deaths worldwide at the time of writing ^1^. Healthcare workers (HCW) may be at greater infection risk compared to the general population ^2– 5^. Many infections are asymptomatic ^6^, therefore surveillance of symptomatic coronavirus disease 2019 (COVID-19) underestimates the infection burden. This has led to calls for regular surveillance of asymptomatic HCWs ^7– 10^, to ensure that health care facilities do not become transmission hot-spots, to protect the workforce and vulnerable patients and to prevent community reseeding.

Most SARS-CoV-2 studies have focused on severe hospitalized COVID-19 cases ^11– 14^. Data are lacking on the host response and biology of asymptomatic or pauci-symptomatic infection as well as the early (pre hospitalisation) stages of disease. This undermines efforts to understand the determinants of disease severity.

We sought to provide a resource to address these gaps by establishing a cohort of HCWs who are well and attending work across selected central London hospitals. We aimed to characterize and quantify the rates of HCW infection (particularly mild or asymptomatic) over the first London COVID-19 pandemic wave, with moderate frequency longitudinal comprehensive sampling before infection and in the weeks to months afterwards. Accordingly, we established the COVID-consortium ( https://covid-consortium.com) and the “COVID-19 Immune Protection and Pathogenesis in Healthcare Worker Bioresource” ( NCT04318314). In this manuscript we: (1) provide a description of the study design, (2) present preliminary results of the baseline visit in the first 400 HCWs (single-centre, between March 23 ^rd^ and 31st 2020) and subsequent 331 (multicentre, from mid-April 2020) – focusing on two different time-points in the epidemiologic curve (just before and after the peak of new daily cases in London), and (3) call for research collaborators wishing to access biological samples in participants across the spectrum of COVID-19 to contribute to a range of prespecified objectives, planned by the consortium https://covid-consortium.com/application-for-samples/.

## Methods

### Study approvals

The study was approved by a UK Research Ethics Committee (South Central - Oxford A Research Ethics Committee, reference 20/SC/0149). All participants provided written informed consent.

### Study participants

Adult (>18 years) hospital HCWs who were fit and well to attend work in any role and across a range of clinical areas, were invited to participate via hospital email, posters, staff meetings, training sessions and participant information leaflets (see https://covid-consortium.com). No other inclusion or exclusion criteria were considered.

### Study design

**Figure 1.**
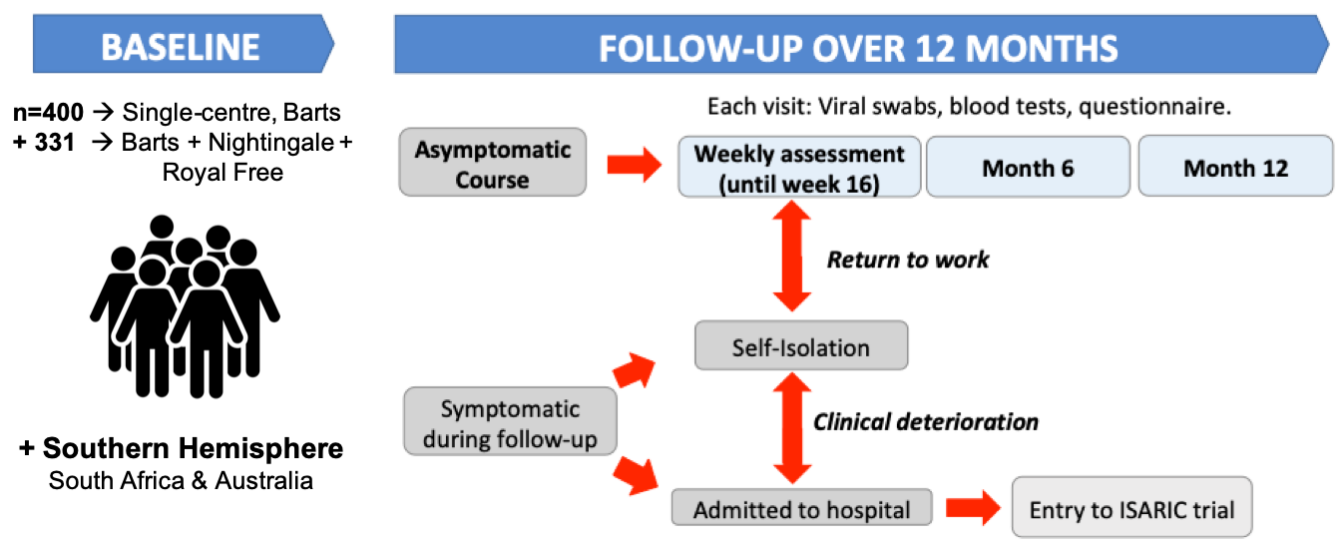
Study flow chart. HCW, healthcare workers.

The “COVID-19 Immune Protection and Pathogenesis in Healthcare Worker Bioresource” ( NCT04318314) uses a prospective cohort design ( Figure 1). The study consists of questionnaires and biological samples (blood samples, nasal swabs ± saliva) performed at all visits: baseline, weekly follow-ups for 15 weeks, and visits at 6 and 12 months.

Recruitment was initially at St Bartholomew’s Hospital, London, UK (400 HCWs recruited between 23 ^rd^ and 31 ^st^ March 2020, just before the peak of new daily cases in London, which happened on the 2 ^nd^ April, with 1,022 new cases confirmed ^15^), a secondary care hospital part of Barts and the Royal London NHS Trust to a local population of 3 million, with specialist cancer and cardiovascular services to a supra-regional population of 6 million. In response to the pandemic, the hospital expanded ventilated intensive care provision for COVID-19 patients to 122 beds across five units.

To improve statistical power for downstream analyses, we expanded the target sample size to n=1,000 and extended recruitment on 17 ^th^ April 2020 (after peak transmission in London, recruitment still ongoing) to other local sites: Nightingale Hospital London (a temporary hospital providing intensive care, set up in response to COVID-19) and Royal Free NHS Hospital Trust (large teaching hospital with specialist expertise in infectious diseases). Collaborations with Cape Town (South Africa) and Sydney (Australia) are also in place to explore the impact of different surge rates, ethnicity, vitamin D levels and the 6-month seasonal difference; unlike UK sites, follow-ups there are performed every fortnight. Our team was comprised of researchers and volunteers from outside of the clinical supply chains.

### Baseline visit

Participants complete a baseline questionnaire ( Table 1) including standard variables related to demographics and exposures. These included occupation, household details, smoking status, physical activity, anthropometry, medical history (including vaccination history, current medication and dietary supplements), occupational exposure (including specific clinical areas and access to/use of personal protective equipment [PPE]), travel history, previous COVID-19 symptoms, proven contact with SARS-CoV-2 infected individuals, and any prior testing for SARS-CoV-2 infection.

**Table 1.** Questionnaire and interview data at baseline assessment.

Questionnaire and interview	
Sociodemographic	Age; sex; ethnicity *; household size; children; postcode
Anthropometric measures	Height; weight
Family history	Family history of coronary artery disease
Health status	Medical history (respiratory, cardiovascular and other diseases); medications; flu vaccine; supplements; pregnancies, miscarriages and contraception; clinical frailty score
Active/ recent exposures/ diseases	Pregnancy; therapies; hospitalization; respiratory infections; symptoms (current and/or previous 14 days); known or perception of having had SARS-CoV-2 infection;
Lifestyle	Smoking; physical activity
Occupational factors	Recruiting centre; department; role; use and perceptions about personal protective equipment; contacts with confirmed COVID-19 patients and/or colleagues; aerosolization procedures; hours worked
Community exposure / environmental factors	Overseas travel; contacts with confirmed COVID-19 cases at home
Psychological factors	General health questionnaire-12; emotional and physical fatigue

* Ethnicity (with specification between brackets also recorded): white (Welsh / English / Scottish / Northern Irish / British Irish / Gypsy or Irish Traveler / any other white background), Asian / Asian British (Indian, Pakistani, Bangladeshi, Chinese, any other Asian background), black / African (African, Caribbean, any other black background), mixed / multiple ethnic groups (white and black Caribbean, white and black African, white and Asian, any other mixed / multiple ethnic background), other ethnic group (Arab, any other ethnic group).

### Follow-up

Following recruitment (baseline visit), if fit and well to attend work, participants would undertake in-person weekly questionnaires using research electronic data infrastructure ( REDCap v8.5.22) ^16^ to capture occupational metadata, new SARS-CoV-2 exposure, symptoms and test results, and biosample collection (blood sampling and nasopharyngeal swabs ± saliva). Following multi-site expansion, information on exercise, pregnancies/contraception, vitamin supplements, working hours and psychological wellbeing (General Health Questionnaire-12 and fatigue questions from the Burnout Assessment Tool) ^17, 18^ were added. The questionnaires used are summarized in Table 2.

**Table 2.** Questionnaire and interview at follow-up assessments.

Questionnaire and interview	
Active/ recent exposures/ diseases	New symptoms; new SARS-CoV-2 infection; new or discontinued medications
Occupational factors	Change in hospital or department/floor; new contacts with confirmed COVID-19 patients and/ or colleagues; new contacts with aerosolization procedures; changes in use and perceptions about personal protective equipment; hours worked
Community exposure / environmental factors	New contacts at home with symptoms and/or confirmed COVID-19
Psychological factors	General health questionnaire-12; emotional and physical fatigue

Subjects who miss an attendance due to shift pattern, redeployment or self-isolation for any reason, resume follow-up on return to work. Illness with suspected COVID-19 is self-reported to the study investigators. Following multisite expansion, participants were also allowed opt-in to a home nasopharyngeal swab and saliva test if self-isolating.

### Sample collection

**Figure 2.**
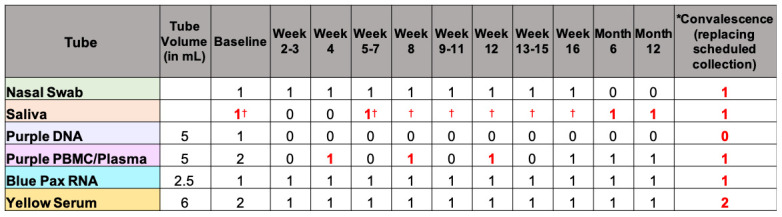
Schedule of biosamples collection. Samples in red: added after ethics amendment on 17/04/2020. * Convalescence visit: first visit after self-isolation with symptoms. † In the first 400 healthcare workers cohort, saliva samples were taken at the first opportunity after week 5; the participants that followed had a saliva sample taken at baseline.

The schedule and quantity of biosample collection is summarized in Figure 2. All study personnel in contact with HCW participants were wearing appropriate PPE in accordance with Public Health England guidance. Nasopharyngeal RNA stabilising swabs are performed at baseline and weekly for 16 weeks. After appropriate training, participants were asked to self-swab both nostrils to minimise the risk to study staff. This strategy was later shown to be reliable when compared to swab collection by health care workers ^19^. Blood samples were collected in Tempus ^TM^ tubes for whole blood RNA, clot activator tubes for serum, and EDTA tubes for plasma, peripheral blood mononuclear cells and DNA ( Figure 2). Following multisite expansion in mid-April, a pool (2–3 mL) of saliva was collected into a dedicated saliva collection tube.

### Initial sample processing

All samples were registered into a Laboratory Inventory Management system onsite and either frozen at -80°C or transferred to a containment level 3 facility. Key samples collected and planned laboratory procedures are described in Table 3.

**Table 3.** List of key samples to be collected and planned laboratory procedures.

Biosample type [Time points]	Processing	Key analyte
**Nasal swab** [Baseline and follow-up until week 16]	Freeze -80ºC	Molecular testing for SARS-CoV-2 ± other pathogens
**Saliva sample** [Baseline/from Week 5 ^*^, convalescence, months 6 and 12]	Freeze -80ºC	Molecular testing for SARS-CoV-2 ± other pathogens
**Yellow serum tube** [Baseline, all follow-ups, convalescence]	Analyse and freeze -80ºC	SARS-CoV-2 antibody testing
**PAX RNA gene tube** [Baseline, all follow-ups, convalescence]	Freeze -80ºC	Transcriptomics
**PAX DNA gene tube** [Baseline]	Freeze -80ºC	Genetics
**Purple EDTA tube** [Baseline, monthly follow-ups, convalescence]	Plasma: centrifuged and stored at -80ºC PBMCs: separated by density gradient centrifugation and cryopreserved	Immunology

* In the first 400 healthcare workers cohort, saliva samples were taken at the first opportunity after week 5; the participants that followed had a saliva sample taken at baseline.

### Core analyses

**Figure 3.**
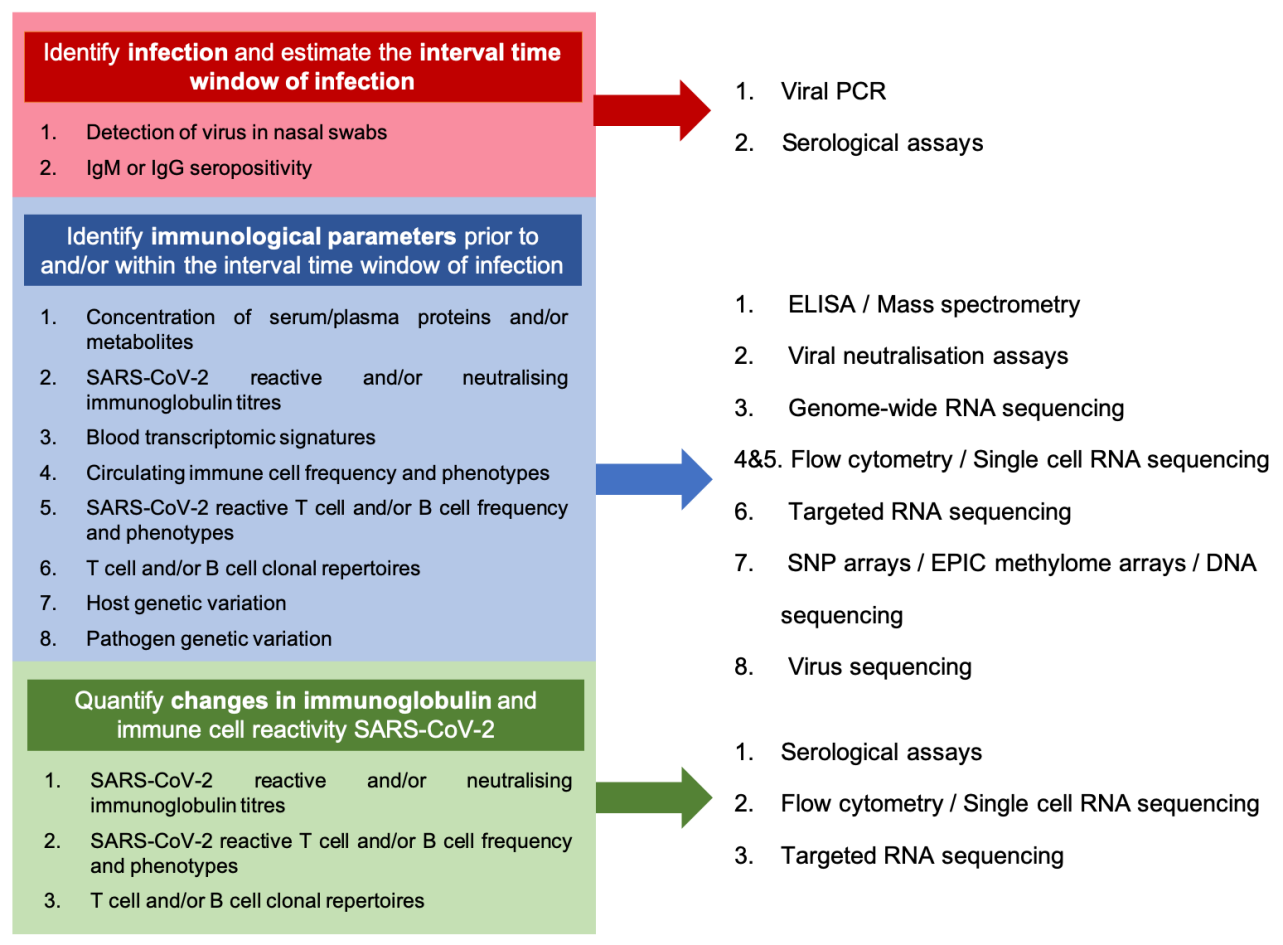
Specific research objectives and summary of experimental approaches.

The following experimental approaches will be implemented ( Figure 3), including:

- Reverse transcription polymerase chain reaction (RT-PCR) of nasal swabs using Roche cobas® SARS-CoV-2 test ^20^.
- Pathogen sequencing with results via the COVID-19 Genomics UK (COG-UK) consortium ^21^.
- Host DNA extraction, quantification and SNP analysis via the Illumina Infinium® GlobalScreeningArray-24v1.0 ^22^.
- IgG antibodies assay to antigen S1, defining seroconversion (initially using the EUROIMMUN assay ^23^).
- Blood RNA extraction focusing on host transcriptomics;
- Peripheral blood mononuclear cells (PBMCs) are a scarce resource and discussions are ongoing about maximising yield;
- Saliva will be diluted and aliquots are available. Further aliquoting will be dependent on demand;
- Other antibody, antigen tests may also be made available should they emerge;
- Serum and plasma will be aliquoted into 100µL samples and divided into packs for individual research teams. Excess RNA (swab and blood) and host DNA will potentially also be available.

### Access procedure

The COVID-19 consortium has developed access systems to facilitate the use of this bioresource by scientists for health-related research of public interest. Research teams can apply to use the bioresource via the study website ( https://covid-consortium.com/application-for-samples/). The access principles are those standard to many bioresources: to maximise yield of timely science, to make results available to other researchers in a reasonable timeframe via a data lake, to reward researchers with appropriate levels of authorship and, where present, intellectual property in a fair, transparent and swift way. We encourage teams to apply and to link their analysis datasets of hospitalised patients with severe disease. We also encourage applications from commercial entities as long as the core principles above apply.

### Statistical analysis

When designing the initial study, we aimed to sample the population prior to exposure. At the time of ethics submission, there was no data to provide precise estimates. The n=400 was pragmatic, aiming for rapid recruitment and limited by logistical challenges of conducting research within a pandemic environment. An initial n=400 was estimated conservatively in order to ensure sampling without compromising selection criteria. Following initial recruitment success, more formal sample size calculation was possible for study expansion and based on an expected average baseline frequency of SARS-CoV-2 infection of 5% in previously undiagnosed HCWs according to studies ^5^. Accordingly, the estimated sample size was n=786 for a β=0.20 and two-sided α=0.05. We targeted a sample size of 1,000 to account for a 20% drop-out rate. However, the specific responses we are seeking are emergent and unknown, and a wider strategy is to link with other studies.

This is a preliminary analysis of the key baseline characteristics of the data. We present discrete variables as absolute frequencies with percentages; continuous normally distributed variables as mean ± standard deviation. Continuous data were checked for normal distribution using Kolmogorov–Smirnov test and visual Q-Q plots assessment. Comparisons between groups were performed using Students’ t-test, while categorical variables were compared using Fisher's exact test. Two-sided p-values <0.05 were considered significant. Statistical analysis was performed using SPSS (version 24.0, IBM Corp., Armonk, NY, USA).

## Results

Baseline characteristics for the first 400 HCWs (single-centre, recruited just before peak transmission, St Bartholomew’s Hospital) and subsequent 331 multicentre study expansion participants (after peak transmission, n=101 in St Bartholomew’s Hospital, n=10 in Nightingale Hospital and n=220 in Royal Free Hospital) are presented in Table 4– Table 6. This reflects all baseline visits between March and May 2020 (total n=731). 

**Table 4.** Baseline demographics and past medical history.

	Single centre (n=400)	Multisite extension (n=331)	Total (n=731)
Age, years	36.7±10.4	39.5±11.4	38.0±10.9
Male, n (%)	157 (39.3)	84 (25.4)	241 (33.0)
BMI, kg/m ^2^	25.0±4.2	25.3±4.6	25.1±4.4
Non-white, n (%)	166 (41.5)	108 (32.6)	274 (37.5)
Household size ≥3 people, n (%)	170 (42.5)	178 (53.8)	348 (47.6)
Children at home, n (%)	147 (36.8)	91 (27.5)	238 (32.6)
Exercise <1.5 hours/week, n (%)	63 (15.8)	60 (18.1)	123 (16.8)
**Past medical history**			
Flu vaccine this season, n (%)	275 (68.8)	234 (70.7)	509 (69.6)
Pregnant in the last 3 months, n (%)	2 (0.5)	1 (0.3)	3 (0.4)
Recent respiratory tract infection, n (%)	73 (18.3)	70 (21.1)	143 (19.6)
Recent surgery, n (%)	6 (1.5)	5 (1.5)	11 (1.5)
Asthma, n (%)	41 (10.3)	37 (11.2)	78 (10.7)
Cancer, n (%)	4 (1.0)	2 (0.6)	6 (0.8)
HIV/Immunodeficiency, n (%)	1 (0.3)	0	1 (0.1)
Rheumatological disease, n (%)	8 (2.0)	1 (0.3)	9 (1.2)
Hypertension, n (%)	26 (6.5)	27 (8.2)	53 (7.3)
Dyslipidaemia, n (%)	18 (4.5)	8 (2.4)	26 (3.6)
Diabetes Mellitus, n (%)	8 (2.0)	7 (2.1)	15 (2.1)
Smoker, n (%)	67 (16.8)	65 (19.6)	132 (18.1)
Family history of CAD, n (%)	54 (13.5)	51 (15.4)	105 (14.4)
Obesity, n (%)	43 (10.8)	54 (16.3)	97 (12.6)

BMI, body mass index; CAD, coronary artery disease; HIV, human immunodeficiency virus. ‘Recent’ refers to the previous 3 months.

**Table 5.** Baseline use of medication and supplements.

Medication/supplement	Single centre (n=400)	Multisite extension (n=331)	Total (n=731)
**Medication**			
ACEI, n (%)	9 (2.3)	11 (3.3)	20 (2.7)
ARB, n (%)	8 (2.0)	4 (1.2)	12 (1.6)
Beta blockers, n (%)	17 (4.3)	4 (1.2)	21 (2.9)
MRAs, n (%)	2 (0.5)	0	2 (0.3)
Aspirin, n (%)	4 (1.0)	2 (0.6)	6 (0.8)
Statins, n (%)	16 (4.0)	11 (3.3)	27 (3.7)
Short-acting inhaled β2 agonist p.r.n., n (%)	21 (5.3)	15 (4.5)	36 (4.9)
Long-acting inhaled β2 agonist, n (%)	6 (1.5)	0	6 (0.8)
Inhaled corticosteroids, n (%)	22 (5.5)	19 (5.7)	41 (5.6)
Oral corticotherapy, n (%)	10 (2.5)	5 (1.5)	15 (2.1)
Use of ibuprofen ≤3 months, n (%)	14 (3.5)	8 (2.4)	22 (3.0)
Ibuprofen use in the past 2 weeks, n (%)	78 (19.5)	54 (16.3)	132 (18.1)
Paracetamol, n (%)	39 (9.8)	14 (4.2)	53 (7.3)
**Supplements**			
Vitamin B complex, n (%)	12 (3.0)	21 (6.3)	33 (4.5)
Vitamin C, n (%)	44 (11.0)	43 (13.0)	87 (11.9)
Vitamin D, n (%)	72 (18.0)	86 (26.0)	158 (21.6)
Multivitamins, n (%)	108 (27.0)	73 (22.1)	181 (24.8)
Iron, n (%)	7 (1.8)	20 (6.0)	27 (3.7)
Zinc, n (%)	14 (3.5)	18 (5.4)	32 (4.4)
Fish oil / omega-3 fatty acids, n (%)	18 (4.5)	21 (6.3)	39 (5.3)

ACEI, angiotensin-converting-enzyme inhibitors; ARB, angiotensin II receptor blockers; MRA, mineralocorticoid receptor antagonists.

**Table 6.** Baseline exposure to SARS-CoV-2 and symptoms.

Variable	Single centre (n=400)	Multisite extension (n=331)	Total (n=731)
**Exposure**			
Contact with confirmed COVID-19 patient, n (%)	116 (29.0)	196 (59.2)	312 (42.7)
Contact with confirmed COVID-19 colleague, n (%)	54 (13.5)	162 (48.9)	216 (29.5)
Confirmed COVID-19 household contact, n (%)	2 (0.5)	6 (1.8)	8 (1.1)
Think had COVID-19, n (%)	26 (6.5)	79 (23.9)	105 (14.4)
Does not wear any form of PPE, n (%)	89 (22.3)	54 (16.3)	143 (19.6)
Oversees COVID-19-care procedure prone to aerosolization, n (%)	94 (23.5)	93 (28.1)	187 (25.6)
Overseas travel in 2020, n (%) ^*^	160 (40.0)	139 (42.0)	299 (40.9)
France, n (%)	25 (6.3)	15 (4.5)	40 (5.5)
Italy, n (%)	11 (2.8)	12 (3.6)	23 (3.1)
Spain, n (%)	24 (6.0)	17 (5.1)	41 (5.6)
**HCW role**			
Doctor, n (%)	83 (20.8)	65 (19.6)	148 (20.2)
Nurse, n (%)	126 (31.5)	102 (30.8)	228 (31.2)
Allied healthcare professional, n (%)	145 (36.3)	56 (16.9)	201 (27.5)
Other, n (%)	46 (11.5)	108 (32.6)	154 (21.1)
**Working place**			
ICU, n (%)	60 (15.0)	81 (24.5)	141 (19.3)
Anaesthesia department, n (%)	9 (2.3)	3 (0.9)	12 (1.6)
Emergency department, n (%)	0	24 (7.3)	24 (3.3)
**COVID-19-like symptoms in the last 14 days, n (%)**	163 (40.8)	86 (26.0)	249 (34.1)
Nasal congestion, n (%)	64 (16.0)	29 (8.8)	93 (12.7)
Odynophagia, n (%)	64 (16.0)	15 (4.5)	79 (10.8)
Productive cough, n (%)	25 (6.3)	3 (0.9)	28 (3.8)
Dry cough, n (%)	42 (10.5)	19 (5.7)	61 (8.3)
Fever, n (%)	24 (6.0)	1 (0.3)	25 (3.4)
Chills/rigors, n (%)	13 (3.3)	4 (1.2)	17 (2.3)
Chest pain, n (%)	13 (3.3)	6 (1.8)	19 (2.6)
Dyspnoea, n (%)	19 (4.8)	8 (2.4)	27 (3.7)
Myalgia, n (%)	21 (5.3)	8 (2.4)	29 (4.0)
Fatigue, n (%)	39 (9.8)	16 (4.8)	55 (7.5)
Diarrhoea, n (%)	14 (3.5)	4 (1.2)	18 (2.5)
Nausea/vomiting, n (%)	4 (1.0)	3 (0.9)	7 (1.0)
Anosmia, n (%)	17 (4.3)	11 (3.3)	28 (3.8)
Ageusia, n (%)	18 (4.5)	7 (2.1)	25 (3.4)

COVID-19, coronavirus disease 2019; HCW, healthcare workers; ICU, intensive care unit; PPE, personal protective equipment. * No HCW reported having travelled to/from mainland China in 2020.

### Demographics

The mean age of all study participants was 38 ± 11 years (0.7% >65 years), 67% female, 37% were black, Asian or minority ethnicities. Demographics are further detailed in Table 4.

### Past medical history and medication

Co-morbidities and COVID-19 risk factors ( Table 4) reported included: 18% smokers, 11% asthma, 7% hypertension, 4% dyslipidaemia, 2% diabetes mellitus; 1% rheumatological disease and one participant with coronary artery disease. Body mass index (BMI) was 25.1 ± 4.4 kg/m ^2^, 97 (13%) participants were obese (BMI >30 kg/m ^2^). The proportion of sedentary participants (reported exercise <1.5 hours/week) was 17%.

Medications are detailed in Table 5. These included short-acting β2 agonist inhalers (5%), inhaled corticosteroids (6%), ACE inhibitors (3%), statins (4%) and paracetamol (7%); 18% of the participants reported having taken ibuprofen in the two weeks prior to recruitment. Over-the-counter supplements rates of usage were: 25% multivitamins, 22% vitamin D and 12% vitamin C.

### Community/social exposure

The proportion of HCWs with a household size of at least three people was 48% (n=348), with a third of the participants reported having children at home ( Table 6). Only eight participants (1%) had a proven contact with a confirmed COVID-19 case at home ( Table 6). Overall, 41% percent (n=299) of HCWs reported having travelled overseas in 2020.

### Occupational exposure

Roles of HCWs ( Table 6) included nurses (n=228), allied health professionals (n=201, e.g. radiographers, cardiac physiologists), doctors (n=148), administrative staff (n=30) and other healthcare roles (n=124, including managers, phlebotomists, laboratory and pharmacy staff, managers, porters, cleaners). HCWs worked in a range of departments ( Table 6) – one-fifth worked in the intensive care unit. The multisite cohort (recruited later in the pandemic wave) reported significantly more contacts with confirmed COVID-19 patients (59 vs 29%, p<0.001) and confirmed COVID-19-positive colleagues (49 vs 14%, p<0.001) at work. They also more frequently reported wearing PPE (84 vs 78%, p=0.039). Overall, 29% (n=210) wore long-sleeve gown, 54% (n=398) plastic aprons, 51% (n=373) surgical masks and 31% (n=230) FFP3 masks; 217 HCWs (30%) expressed concerns about PPE being insufficient or inadequate and 81 (11%) stated that PPE policy is either confusing or unclear.

### Symptoms, infection and serology

The prevalence of COVID-related symptoms in the two weeks prior to recruitment was 34% (n=249/731), significantly higher in the early cohort recruited in March (41% vs late cohort 26%, p<0.001). More HCWs from the multisite cohort recruited at the later time point thought that they had had prior COVID-19 (24 vs 7% in the initial cohort, p<0.001). Overall, the most prevalent symptom was nasal congestion (13%), followed by odynophagia (11%), dry cough (8%) and fatigue (8%). A recent (<3 months) respiratory tract infection was reported in 20% of the participants.

**Figure 4.**
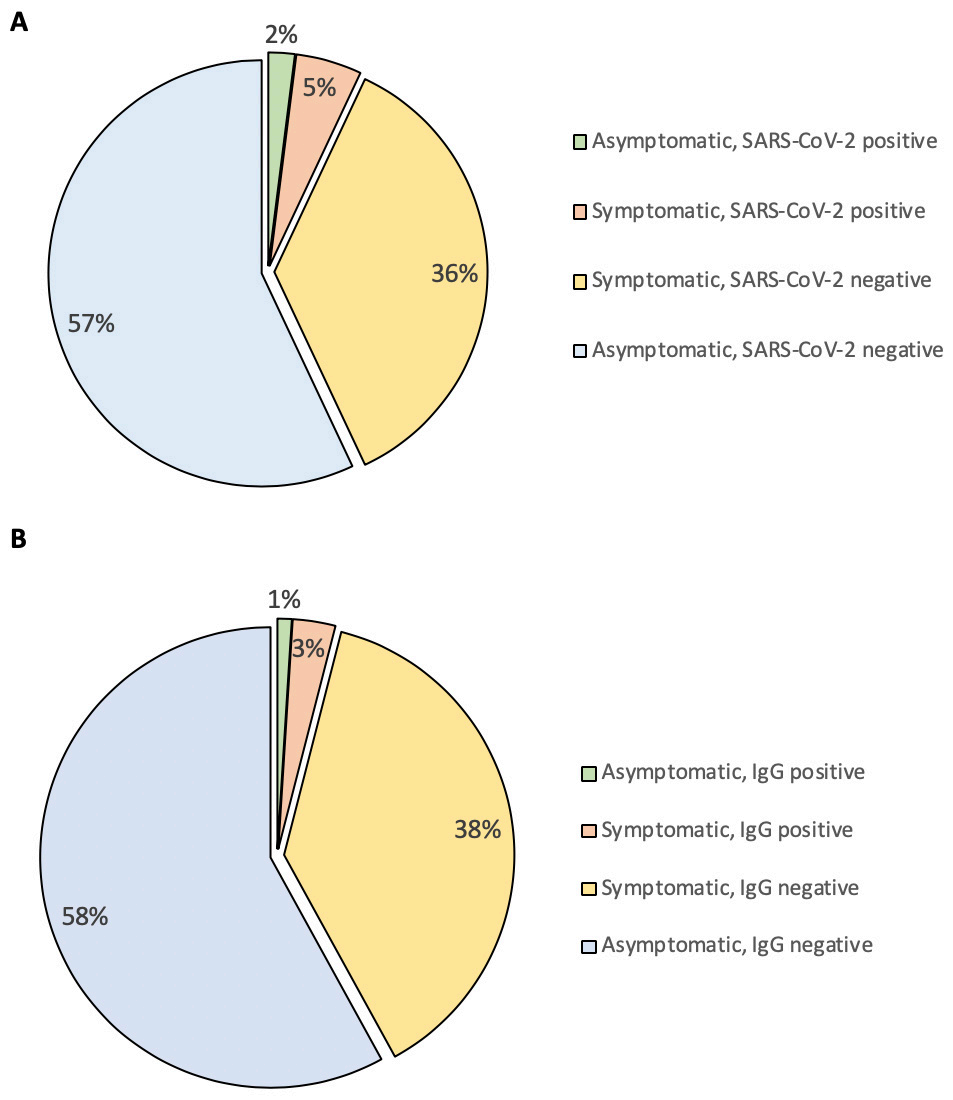
Symptoms in the 14 days before recruitment. ( **A**) SARS-CoV-2 test results, and ( **B**) serology results.

SARS-CoV-2 RT-PCR and serology were processed for the first cohort (n=400) and are described. PCR for SARS-CoV-2 was positive in 28 of 396 (7.1%, 95% confidence interval 4.9 – 10.0%, 4 swabs not available), of which 20 (71%) were symptomatic in the 14 days before ( Figure 4A). In the same cohort, 15 of 385 (3.9%, 2.4 – 6.3%) had IgG positive serology to S1 spike protein, of which 11 (73%) reported prior symptoms ( Figure 4B) and 3 (20%) were PCR positive. A total of 40 participants (10%) were PCR and/or IgG positive at baseline.

## Discussion

This study is establishing a bioresource (COVID-consortium) derived from health care workers, with samples taken at the time of pre‑symptomatic incident infection, linked to data on clinical outcomes, serology and follow-up sampling to evaluate the quality and duration of immune memory to the virus. Here we present preliminary baseline data on the first 731 participants, comprised of a single-centre cohort recruited in March 2020 just before the time of peak community transmission in London, and a subsequent expanded multicentre cohort recruited from mid-April 2020. This resource should enable collaborative science and approved investigators can apply for sample access or access to the resultant data lake to address specific questions or for incorporation into larger COVID-19 datasets.

### HCWs baseline characteristics

SARS-CoV-2 can rapidly spread to patients and HCWs in hospitals, and HCWs generally have been particularly hard hit with high reported rates of infection ^2, 3, 5, 24^. Our cohort is representative of a multi-ethnic urban UK population of working age, and more specifically of the NHS workforce across different clinical roles and departments. Confirmed COVID-19 contacts were low in the community (1%), but much higher in-hospital (43% patients, 30% colleagues), particularly in the second cohort (recruited later). All participants were self-reported as fit to attend work on all clinical visits, and at baseline the majority of participants had been asymptomatic and did not think that they had been infected. Nevertheless, 1 out of 10 participants had a confirmed baseline SARS-CoV-2 infection confirmed by PCR and/or positive serology test that could represent current or previous infection, at the beginning of peak transmission in March.

Of interest, two different timepoints are presented here. As one would expect, the proportion of HCWs who reported prior symptoms was significantly higher in participants recruited just before peak community transmission ^25^, and those recruited a month later more often reported they suspected that they had already had COVID-19.

### The COVID-19 bioresource

The scientific community has merged forces to tackle this unprecedented pandemic. Since the start of January 2020 (until 31 ^st^ May 2020), 160 research projects on COVID-19 received a favourable opinion by the UK NHS Health Research Authority (last updated list on 3 ^rd^ June 2020), the majority focused on confirmed COVID-19 patients ^6^. Emergent studies are now targeting mild and population disease, but almost all missed peak transmission. Larger-scale community surveillance studies typically also do not have temporal granularity to detect early disease changes and may be more focused on providing data to improve modelling rather than host:pathogen biology. Studying HCWs is a middle ground - subjects are deemed fit to work, but have higher exposure rates to confirmed COVID-19 cases, and can also be frequently assessed. COVID-19 bioresources in the general population and HCWs have already been established. Studies such as the COVID-19 Emergency Response Assessment (CERA) ^26^ and the Rapid European SARS-COV-2 Emergency research Response (RECOVER) ^27^ use qualitative instruments to assess the physical and psychological well-being of frontline doctors at different phases of the pandemic. The SARS-CoV-2 Acquisition in Frontline Health Care Workers – Evaluation to Inform Response (SAFER) ^28^ study will perform qualitative interviews and collect nose and throat swabs twice weekly and serum samples monthly from healthcare staff. Preliminary results from the SAFER study revealed a higher PCR positive rate of 21% among HCWs (42/200), but during the whole first month of observation (starting between 26 ^th^ March and 8 ^th^ April 2020) ^29^. The COVID-19 Staff Testing of Antibody Responses Study (CO-STARS) follows a similar design, with serologies performed monthly for 6 months and then 6-monthly for a total of 6 years ^30^.

The comprehensive (questionnaires and biosamples) serial assessment of asymptomatic participants starting just before peak community transmission of SARS-CoV-2 makes our bioresource a precious dataset for the scientific community. We expect that the data sampled from HCWs facilitates understanding of mild disease and subclinical infection at a more rapid rate than the general population allowing comparison with those more severely affected or hospitalised for COVID-19. The COVID-19 consortium ( https://covid-consortium.com) and the “COVID-19 Immune Protection and Pathogenesis in Healthcare Worker Bioresource” ( NCT04318314) thus encourages research teams to apply and even potentially link their own datasets to ours (with results expected to be returned to the data lake for collaborative science). Some of the fields worth exploring include immune responses during the subclinical phases of infection, properties of the immunoglobulins and immune cellular reactivity (correlations between viral RNA PCR and subsequent serology, persistence of neutralizing antibodies, immune decay and longevity of serological responses), host and viral genetic variation, and other environmental or acquired risk factors.

### Limitations

The three centres initially included reflect the epidemiological curve of a single city (London). The COVID-19 bioresource started at peak community transmission with prevalent asymptomatic infection in 7.1% and seropositivity of 3.8% at baseline. In data from the subsequent four weeks, we have already reported that the incident asymptomatic infections fell in line with reductions in the London wide incidence ^31^. Nationwide data are accruing to assess the generalisability of our findings, but there are also opportunities to expand geographical coverage of our bioresource through collaborations with other studies that include serial sampling of HCWs. Although our cohort is ethnically diverse (37% non-white), the frequency of comorbidities is relatively low, there are no children and elderly subjects are under-represented. In addition, our cohort of hospital HCWs is unlikely to be generalisable to other institutional settings such as care homes, or to the wider population given that all participants are of working age and in good general health. A selection bias could also be present as HCWs who felt at higher risk of exposure could have been more motivated to participate in the study than the rest of the hospital staff. However, it is worth highlighting that participants had to sign consent forms recognising that they would not receive results in real-time (particularly when access to tests was limited early in the pandemic), and therefore it should have minimised bias in recruitment.

## Conclusions

Just before the peak of COVID-19 infections in the UK we established a rich and granular bioresource of healthcare workers with the aim of gathering insights into early disease / asymptomatic SARS-CoV-2 infection. Combining exposure with multi-qualitative and quantitative assessments, we envision a more complete picture of immune response in this context. The samples and data securely curated this bioresource are now accessible to the wider scientific community by application.

## Data availability

The COVID-19 consortium has developed access systems to facilitate the use of this bioresource and the data underlying this article by scientists for health-related research of public interest. However, although participants are pseudoanonymsed, there is data regarding home addresses, household characteristics, and other details that could potentially lead to identification. Research teams can therefore apply to use the bioresource via the application form that can be found on the study website ( https://covid-consortium.com/application-for-samples/).
