# Supplementary material for: Healthcare Workers Bioresource: Study outline and baseline characteristics of a prospective healthcare worker cohort to study immune protection and pathogenesis in COVID-19
Source: Wellcome Open Res. 2020 Oct 12;5:179. Originally published 2020 Jul 28. [Version 2] doi: 10.12688/wellcomeopenres.16051.2 (PMC7836029; doi:10.12688/wellcomeopenres.16051.2)
Supplement: Peer review history

# Peer review: Reviewer response for version 2

**DOI:** 10.21956/wellcomeopenres.17993.r41916

**Published:** 2021-01-22

**Authors:** Berta Grau-Pujol

**Affiliations:** 1 Barcelona Institute for Global Health (ISGlobal), Hospital Clínic - Universitat de Barcelona, Barcelona, Spain

The study is clearly described, as well as the changes occurred in study procedures. The emerging COVID-19 pandemic and availability of tests and materials during this period justifies these changes.  Although it provides baseline information, it is worth sharing it, and thus indexing it. 

 Few comments on Methodology: 

- It is not described which statistical power they had. 
- Study design and procedures in Cape Town and Sydney should be further described. 
- In core analysis, the authors use future tense, not sure that is correct. 
- Further statistical analysis could be conducted, such us regression analysis. Participants' site could be also considered.

Is the work clearly and accurately presented and does it cite the current literature?

Yes

If applicable, is the statistical analysis and its interpretation appropriate?

Partly

Are all the source data underlying the results available to ensure full reproducibility?

Yes

Is the study design appropriate and is the work technically sound?

Yes

Are the conclusions drawn adequately supported by the results?

Yes

Are sufficient details of methods and analysis provided to allow replication by others?

Partly

Reviewer Expertise:

Epidemiology of infectious diseases.

I confirm that I have read this submission and believe that I have an appropriate level of expertise to confirm that it is of an acceptable scientific standard, however I have significant reservations, as outlined above.

# Peer review: Reviewer response for version 1

**DOI:** 10.21956/wellcomeopenres.17607.r40258

**Published:** 2020-09-24

**Authors:** Aodhan Breathnach

**Affiliations:** 1 Infection Care Group, St George's University Hospitals NHS Foundation Trust, London, UK

This is obviously a very preliminary description of a just-started observational study, with no results reported other than the baseline characteristics of the patients. The stated purpose - to prospectively study a cohort of HCWs, thereby including milder and earlier COVID19 infections that would not necessarily be hospitalised - is worthwhile. The main value of reporting at this stage seems to be to publicise the study and to invite collaborators.

 The paper is clear and well-organised. The limitations are acknowledged, the main one being that the cohort of HCWs  is of necessity a working-age population, with few elderly workers, and no children.

 There is a typo in the last paragraph, and the last sentence of the paper does not make complete sense.

 The link to the Covid Consortium application form is given three times in the last page - is this repetition necessary?

 The paper is well referenced, though I was unable to find Ref 25 online.

Is the work clearly and accurately presented and does it cite the current literature?

Yes

If applicable, is the statistical analysis and its interpretation appropriate?

I cannot comment. A qualified statistician is required.

Are all the source data underlying the results available to ensure full reproducibility?

Yes

Is the study design appropriate and is the work technically sound?

Yes

Are the conclusions drawn adequately supported by the results?

Partly

Are sufficient details of methods and analysis provided to allow replication by others?

Yes

Reviewer Expertise:

I am a clinical microbiology consultant in a combined Infection Department. As such, my academic work is limited to observational and epidemiological research, with an interest in bacteraemia, endocarditis, infection control and OPAT services. I have recently become involved in analysing COVID infections and seroconversions in our hospital staff.

I confirm that I have read this submission and believe that I have an appropriate level of expertise to confirm that it is of an acceptable scientific standard.

# Response

**Published:** 2020-10-05

**Authors:** João Augusto

We thank the reviewer for the pertinent comments.

We provide below a point-by-point response.

**There is a typo in the last paragraph, and the last sentence of the paper does not make complete sense.**

Thank you. This has now been corrected.

**The link to the Covid Consortium application form is given three times in the last page - is this repetition necessary?**

Thank you. We agree and have now amended this.

**The paper is well referenced, though I was unable to find Ref 25 online.**

We have now added a direct link to the UK COVID-19 surveillance reports.

# Peer review: Reviewer response for version 1

**DOI:** 10.21956/wellcomeopenres.17607.r39943

**Published:** 2020-09-01

**Authors:** David Murdoch

**Affiliations:** 1 Department of Pathology and Biomedical Science, University of Otago, Christchurch, New Zealand

This paper presents the design and baseline characteristics of a prospective cohort study of healthcare workers (HCWs) for use as a "bioresource" for research on COVID-19. The study population has a high prevalence of exposure to COVID-19 and evidence of recent SARS-CoV-2 infection.

 Specific comments:

1. The study population is a relatively select group of healthcare workers. It is essential that this group is described in greater detail in order to best understand the generalisability of any study findings. As such, it is unclear whether any of the HCWs do not have direct patient contact (e.g. laboratory staff). It would also be helpful to have a commentary on whether there is any selection bias among those volunteering to be study participants, beyond the baseline data presented. This is critical in order to understand any study population-level analyses.
2. A cohort study is an observational study by definition. Therefore "observational" is an unnecessary adjective in several places.
3. What definition of ethnicity is being used? Is the same definition being used for different countries?

Is the work clearly and accurately presented and does it cite the current literature?

Yes

If applicable, is the statistical analysis and its interpretation appropriate?

Yes

Are all the source data underlying the results available to ensure full reproducibility?

Yes

Is the study design appropriate and is the work technically sound?

Yes

Are the conclusions drawn adequately supported by the results?

Yes

Are sufficient details of methods and analysis provided to allow replication by others?

Yes

Reviewer Expertise:

Infectious diseases, microbiology, epidemiology.

I confirm that I have read this submission and believe that I have an appropriate level of expertise to confirm that it is of an acceptable scientific standard, however I have significant reservations, as outlined above.

# Response

**Published:** 2020-10-05

**Authors:** João Augusto

We thank the reviewer for the time taken to review our manuscript and for the pertinent comments. We provide below a point-by-point response.

**1. The study population is a relatively select group of healthcare workers. It is essential that this group is described in greater detail in order to best understand the generalisability of any study findings. As such, it is unclear whether any of the HCWs do not have direct patient contact (e.g. laboratory staff). It would also be helpful to have a commentary on whether there is any selection bias among those volunteering to be study participants, beyond the baseline data presented. This is critical in order to understand any study population-level analyses.**

Thank you. This cohort represents an heterogenous group with different degrees of patient contact and consequently different degrees of exposure – from nurses/doctors to laboratory/pharmacy staff and managers. We, however, believe that this can prove advantageous to understand risk factors. A more detailed description of the HCWs roles is now provided in the results section. The characteristics here described are likely generalisable to HCWs in most institutions - participants recruited across all staff groups with broad baseline demographics. They are not, however, generalisable to the wider population given that all participants are of working age and in good general health. More granular data on exposure will be part of the study investigation and presented in subsequent publications Of note, the factor “patient contact” was taken into account in our questionnaire (with details of the healthcare role, hospital department/unit, and COVID-care procedure - if any). 

We acknowledge a selection bias could be present as HCWs who felt at higher risk of exposure could have been more motivated to participate in the study than the rest of the hospital staff. However, it is worth highlighting that participants had to sign consent forms recognising that they would not receive results in real-time (particularly when access to tests was limited early in the pandemic), and therefore it should have minimised bias in recruitment. This has now been added to the limitations section. 

**2. A cohort study is an observational study by definition. Therefore "observational" is an unnecessary adjective in several places.**

Thank you. We agree and have now corrected this.

**3. What definition of ethnicity is being used? Is the same definition being used for different countries?**

We have now clarified this - see legend of table 1.
